# Feasibility and outcomes of supplemental gait training by robotic and conventional means in acute stroke rehabilitation

**DOI:** 10.1186/s12984-023-01243-3

**Published:** 2023-10-04

**Authors:** Mukul Talaty, Alberto Esquenazi

**Affiliations:** 1 Gait and Motion Analysis Laboratory, MossRehab, Elkins Park, PA 19027 USA; 2 Penn State University, 1600 Woodland Road, Abington, PA 19001 USA

**Keywords:** Stroke, Gait, Lokomat®, Inpatient Rehabilitation Facility

## Abstract

**Introduction:**

Practicality of implementation and dosing of supplemental gait training in an acute stroke inpatient rehabilitation setting are not well studied but can have positive impact on outcomes.

**Objectives:**

To determine the feasibility of early, intense supplemental gait training in inpatient stroke rehabilitation, compare functional outcomes and the specific mode of delivery.

**Design and setting:**

Assessor blinded, randomized controlled trial in a tertiary Inpatient Rehabilitation Facility.

**Participants:**

Thirty acute post-stroke patients with unilateral hemiparesis (≥ 18 years of age with a lower limb MAS ≤ 3).

**Intervention:**

Lokomat® or conventional gait training (CGT) in addition to standard mandated therapy time.

**Main outcome measures:**

Number of therapy sessions; adverse events; functional independence measure (FIM motor); functional ambulation category (FAC); passive range of motion (PROM); modified Ashworth scale (MAS); 5 times sit-to-stand (5x-STS); 10-m walk test (10MWT); 2-min walk test (2MWT) were assessed before (pre) and after training (post).

**Results:**

The desired supplemental therapy was implemented during normal care delivery hours and the patients generally tolerated the sessions well. Both groups improved markedly on several measures; the CGT group obtained nearly 45% more *supplemental* sessions (12.8) than the Lokomat® group (8.9). Both groups showed greater FIM improvement scores (discharge – admission) than those from a reference group receiving no supplemental therapy. An overarching statistical comparison between methods was skewed towards a differential benefit (but not significant) in the Lokomat® group with medium effect sizes. By observation, the robotic group completed a greater number of steps, on average. These results provide some evidence for Lokomat® being a more efficient tool for gait retraining by providing a more optimal therapy “dose”.

**Conclusions:**

With careful planning, supplemental therapy was possible with minimal intrusion to schedules and was well tolerated. Participants showed meaningful functional improvement with relatively little supplemental therapy over a relatively short time in study.

**Supplementary Information:**

The online version contains supplementary material available at 10.1186/s12984-023-01243-3.

## Introduction

Stroke is a major cause of impaired trunk control and gait disability. [1–3] Due to the altered supraspinal control, the abnormal gait pattern post-stroke may be the result of muscle weakness, spasticity and abnormal motor control. [4] Impaired walking ability not only reduces the functional independence of stroke survivors, but also affects quality of life and increases the risk of falls. [5, 6] Improving walking function is often a key component of the post-stroke rehabilitation program. Rehabilitation based on the concepts of repetitive, intensive, task-oriented training has been shown to be effective. [7, 8] Motor learning reflects a neural specificity of practice since motor skill acquisition involves the integration of the sensory and motor information that occurs during practice, and ultimately, leads to the sensorimotor solution that results in accurate, consistent and skillful movements. [9] In addition to quality, other key components of efficient and maximal recovery are timing, intensity and engagement of the rehabilitation intervention[10–13].

It is well established by both animal [14–16] and human studies [16–18] that the greatest recovery post-stroke occurs within the first three months. This emphasizes the need for early rehabilitative intervention approaches to improve balance and mobility in this population [16, 17]. In addition to starting early, while more exercise is also generally good, aspects of the optimal dose are not clear. Some key open questions regarding optimal therapy dosing are: (1) how much early extra therapy is practical to be delivered during acute rehabilitation? (2) is this well tolerated by patients? and (3) does delivery mode matter? We aimed to provide knowledge related to the volume of the optimal dose by comparing the outcomes of a robotic vs. a conventional therapist-driven supplemental early post-stroke gait training. The primary objective in this assessor-blinded, randomized controlled trial was to determine the feasibility of supplemental gait training in an inpatient rehabilitation facility (IRF) that requires at least three hours of daily therapy. Secondarily, we sought to compare the outcomes of two gait training modalities, a robotic (Lokomat®) and conventional gait training (CGT) techniques.

## Methods

### Study design and randomization

**Fig. 1:**
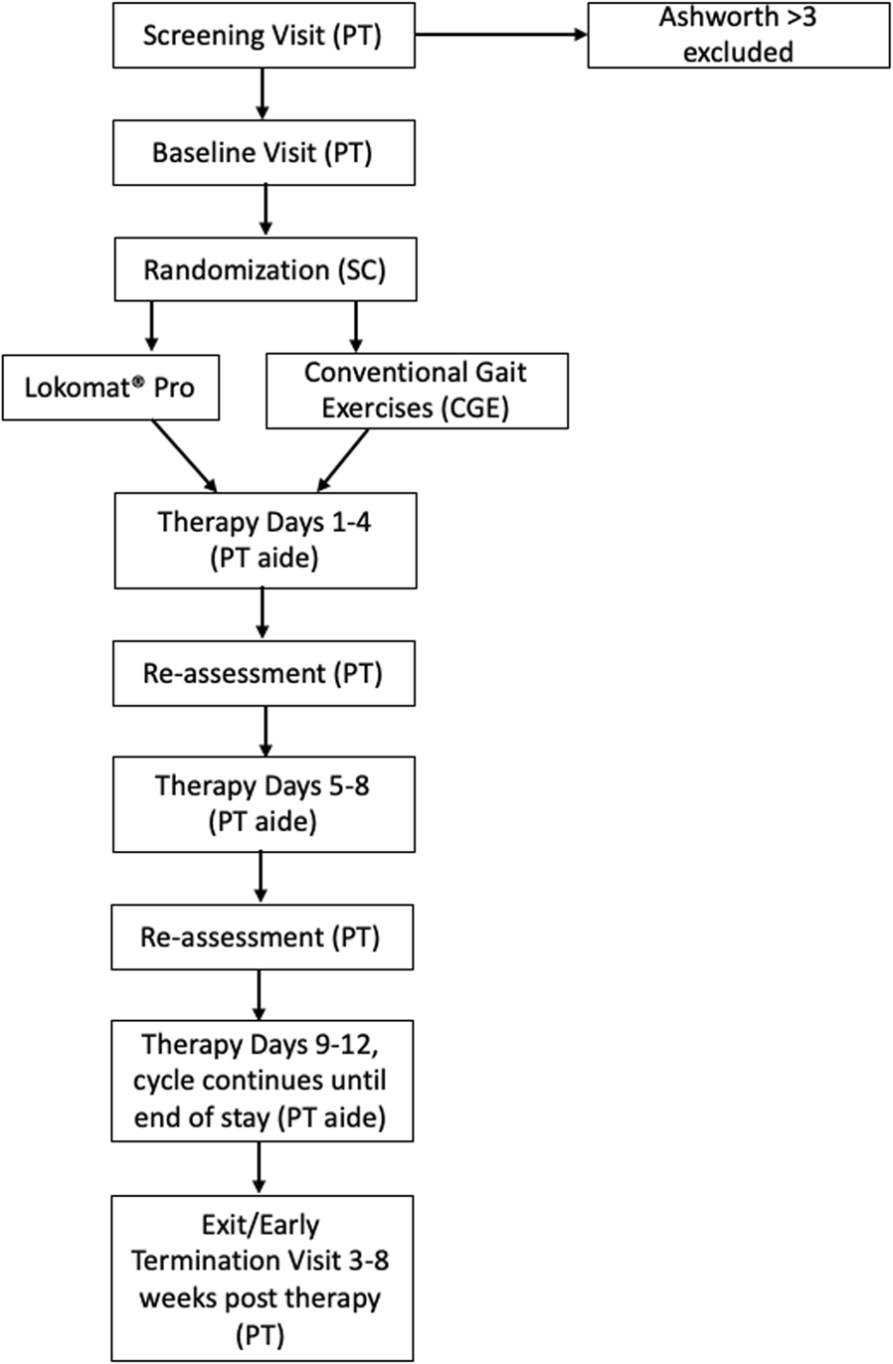
Study design overview. Abbreviations used were *PT* Physical Therapist, *SC* Study Coordinator

This assessor blind, randomized feasibility and pilot study was conducted in a tertiary IRF. The study protocol was approved by the local Institutional Review Board (IRB). Figure 1 provides an overview of the study design. Assessments were performed before (pre) intervention, and at least once a week based on the length of stay (LOS) and at discharge (post) by trained assessors who were blinded to group allocation. All assessors and assessments were performed on a different care unit than the intervention to ensure blinding. Allocation of intervention was determined by a simple randomization sequence using Microsoft Excel. A research coordinator enrolled participants, generated the random allocation sequence and assigned and scheduled participants to interventions. The first 30 participants who qualified and agreed to participate were enrolled into the study and were placed into the groups following the above noted randomization scheme.

### Participants

Patients with a recent stroke (on average, less than three weeks post-injury), admitted to an IRF were assessed for eligibility by their treating clinical team (physiatrist and/or physical therapist) between April 2017 and July 2019. Inclusion criteria were: (1) Adult (≥ 18 years of age) after first stroke; (2) able to tolerate ≥ 12 minutes of upright position using a tilt table, standing frame, body weight support (BWS) harness or assistance; (3) medical stability and cardiorespiratory status sufficient to tolerate aerobic exercise protocol. Exclusion criteria were: (1) MAS score 4 in ankle, knee or hip joints; (2) other neurological injury or disorder affecting the central nervous system; (3) previous lower limb peripheral nerve injury; (4) lower limb joint contractures that interfere with walking; (5) open skin lesions or anatomic deformities in the lower limbs and/or torso that may interfere with the Lokomat® exoskeleton or support harness application; (6) bone problems (non-consolidated fractures; unstable spine; and severe osteoporosis with history of fractures; (7) severe cognitive deficits; (8) recent cardiac or active pulmonary disease, labile blood pressure; (9) severe vascular disorders of the lower limbs; (10) scheduled elective surgery or other procedures during the study; (11) uncontrolled seizures; (12) pregnancy. All participants gave written informed consent before start of the study. Demographic and clinical characteristics collected included: sex, hemiparetic side, age, stroke type (hemorrhagic/ischemic), stroke onset date, LOS, days from informed consent form (ICF) signature to discharge and number of completed training sessions. The trial took place in an Inpatient Rehabilitation Facility (IRF) to which patients were transferred after acute care on day 4 to 10 post stroke. For this study, LOS refers to time in the IRF only. The therapist perceived exertion during delivery of supplemental training for both groups was documented using the modified RPE (Borg Scale).

### Interventions

Lokomat® or conventional gait training (CGT) was provided in addition to the mandated three hours of conventional IRF therapy. Both groups received up to four 45-min individual gait training sessions per week. Treatment was well defined and standardized at the top level to the degree possible given the individualized nature of gait training. In each group, the protocol was focused on achieving the maximum number of steps as possible and active participation during the training time. How the therapist achieved this was based, in part, on walking ability for each participant. An overview of the structure and priority of activities and progression is discussed for both groups in the below paragraphs; additional details are provided in the Additional file 1: Appendix S1. Vital signs were collected at the start of each training session. Rest periods were provided if needed.

#### Lokomat®

The Lokomat® exoskeleton is comprised of two actuated hip and knee leg orthoses that are attached to the participant’s limbs and torso by cuffs and straps. Spring assisted ankle dorsiflexion is implemented via a strap over the shoe. The orthoses, size and position of leg cuffs were measured and adjusted to the participant ensuring that walking in the device was as natural and comfortable as possible. Individually tailored levels of body weight support and walking velocity were determined for the Lokomat®. For the present study, the level of guidance force during the Lokomat® training was set to 50%, which allowed for small deviations and required more active patient participation compared to fully guided walking training. At subsequent training sessions, a therapist made individual determinations about adjustments to speed (increases) and BWS (decreases) based on the participant’s performance – with the goal of increasing active participation time (i.e. decreasing rest time) and increasing overall number of steps taken.

#### Conventional

The conventional gait training (CGT) consisted of therapist assisted and/or use of assistive device (walker) for supported walking, if participants were able, or pre-walking exercises (trunk balance, sitting/standing balance, leg movements, etc.) if participants were not able to walk. In general, therapy was progressed with the aim to have participants actively participating for 45 minutes and to maximize steps walked.

Treatment was provided by three therapists for the CGT group and a different three for the Lokomat® group. The “regular therapy team” was aware that their patients were involved in a trial (as they could see their patients’ schedule of activities) but were blinded to which of the two arms (Lokomat® or CGT). Paramount to this, the rating team was blinded in the same fashion and that team came from a different unit so there was no possibility for unblinding. Several approaches were used to assess fidelity with which the protocol was implemented. Especially since this was a pilot trial, careful attention was paid in the early stages to ensure key objectives were able to be accomplished. For example, the research coordinator worked diligently to try to ensure all supplemental sessions could be implemented during a busy inpatient schedule and with the normal complement of therapist during regular treatment hours. The therapists were trained by the research staff on the key goals of the supplemental sessions and how to go about achieving them. Consistency of the specific training, beyond the objectives and priorities as outlined in the supplemental material provided, was not enforced as one goal of the study was to implement supplemental *clinically motivated training*. The exact means by which the training was implemented were not designed to be standardized beyond that framework. Data were regularly monitored by the research assistant and/or investigator(s) to confirm they were being collected properly and completely. Feedback was provided to the treating teams as needed.

### Outcomes

#### Primary outcome measures

Feasibility of implementation, tolerability, number of training sessions completed, serious adverse events (SAEs).

#### Secondary outcome measures

Functional independence measure (FIM) and FIM efficiency, functional ambulatory category (FAC), passive range of motion (PROM), Modified Ashworth Scale (MAS), 10-meter walk test (10MWT), 2-minute walk test (2MWT), 5 Times Sit to Stand (5x-STS) and the Modified Borg Scale were collected to compare outcomes between the training groups and compared to standard of care only group that was obtained from our database. Participants were allowed to use an assistive device for the test if required. Secondary outcome measures were collected at baseline, at least once every week and at discharge from the IRF stay.

### Statistical analysis

The Wilcoxon signed-rank (WSR) test and effect size (Cohen’s d) were used to compare the within group differences in pre- and post-training on the outcome measures. A Bonferroni correction *p*-value of 0.005 (0.05/10 – for the ten variables listed in Table 2) was considered as significant. The non-parametric WSR test was used due to the potential sensitivity in estimating normality of residuals in small data sets; t-test were run as a “*safety*” check. A-priori hypotheses for all variables were that they would improve in the post compared to the pre-condition; two-tailed p-values were reported only for knee and ankle MAS and PROM which had the potential to worsen as well as improve. A single factor MANOVA on *change scores* (i.e. post – pre training) on all interval variables (5xSTS, 2MWT, 10MWT, and ankle and knee PROM) was conducted to assess whether there was a difference between the treatment modalities. Pairwise post-hoc ANOVAs were used to identify differences in individual variables. Due to large observed differences in some outcome measures between groups, and the potential confound of varying doses of supplemental training, we performed ANCOVA analyses on the functional variables to control for the dose differential. First, the correlation of each functional outcome measure with a larger effect size (change in FIM, FAC, 10MWT, 2MWT and 5xSTS) to the number of supplemental training sessions was checked, to minimize dilution of the power of the regression testing. Variables that were significantly correlated were subsequently assessed by ANCOVA. Finally, to further assess the effect of the supplemental training (compared to standard of care), we generated a reference group of patients who did not receive supplemental gait training. We extracted FIM data from an outcomes database (eRehabData®, AMPRA Washington, D.C.) of all patients between the ages of 40 and 60 years, admitted to our hospital for stroke from an overlapping period during which the study participants were recruited. Statistical analyses were performed using the Real Statistics toolkit for Microsoft Excel (https://www.real-statistics.com/, Charles Zaiontz).

## Results

Demographic data are shown in Table 1, changes in functional outcomes in Table 2. No SAEs were reported in either group; four CGT participants had AEs that were unrelated to the study. FAC, FIM, 5xSTS, 2MWT and 10MWT significantly improved at discharge (p-values < 0.005) in the Lokomat® group whereas all the same except for 5xSTS were significantly improved for the CGT group. In all but two of the 20 individual pre vs. post comparisons reported in Table 2, the t-test p-values were consistent with those from WSR, and in those two cases, the t-test was more conservative. Furthermore, FAC, FIM, 5xSTS, 2MWT and 10MWT all showed large effect sizes (>0.8) in both Lokomat® and CGT groups, and RPE showed a medium effect size (between 0.6 and 0.8) in the Lokomat® group. Neither the univariate MANOVA analysis to compare the five interval scale outcome variables between modalities (CGT vs. Lokomat®, p=0.21, Pillai Trace) nor any of the associated post-hoc ANOVAs to compare individual differences were statistically significant (last row, Table 2).

**Table 1:** Demographic and clinical features of Lokomat®, the conventional (CGT) and reference group

Characteristics	Lokomat (n = 15)	Conventional (n = 15)	Standard of care (n = 415)
Mean age ± SD (yrs)	63.2 ± 10.0 (42–84)	53.7 ± 16.8 (29–84)	53.1 ± 5.1
Gender (male/female)	12/3	10/5	249/166
Stroke side (left/right)	7/8	11/4	N/A
Stroke type (hemorrhagic/ischemic)	4/11	4/11	62/353
Mean stroke onset ± SD (days)	17.0 ± 9.9 (4–40)	16.9 ± 12.9 (5–46)	N/A
Mean length of stay ± SD (LOS, days)	31.7 ± 13.1 (13–55)	35.3 ± 17.9 (14–70)	N/A
Mean time in study (TS, days)	21.2 (8–35)	27.5 (9–67)	N/A
Mean number of supplemental training sessions ± SD	8.3 ± 4.8 (4–18)	12.0 ± 8.7 (3–33)	0 ± 0 (N/A)

Data shown: mean ± standard deviation (range).

These data include all participants who enrolled in study and reference data (last column). *SD* standard deviation

**Table 2:** Changes in outcome variables from admission to discharge and between treatment modality

	FAC	FIM	Ank. MAS	Knee MAS	5xSTS (sec)	2MWT (m)	10MWT (m/s)	RPE	Ank. DF (deg)	Knee Ext. (deg)
Mean Loko	1.2	24.5	0.3	0.0	− 24.9^a^	25.8	0.24	− 0.7	4.4	1.4
Stdev Loko	1.0	10.5	0.7	1.1	26.7	28.6	0.24	1.1	10.0	3.9
p-value Loko WSR (t-test)	**0.0039** **(0.0012)**	**0.0025** **(2.92E-06)**	0.32 (0.22)	0.94 (1.00)	0.011 (0.0039)	**0.0039** **(0.0049)**	**0.0039** **(0.0026)**	0.049 (0.027)	0.77 (0.92)	0.25 (0.21)
Cohen’s d	**1.1**	**2.3**	0.4	0	**0.9**	**0.9**	**1.0**	**0.6**	0.0	0.4
Mean CGT	1.4	26.6	0.4	0.2	− 9.2^a^	39.5	0.23	− 0.7	1.3	− 0.3
Stdev CGT	1.4	11.0	0.8	0.9	7.7	52.2	0.29	2.4	20.8	2.3
p-value CGT WSR (t-test)	**0.0024** **(0.0018)**	**1.2E-04** **(7.9E-07)**	0.094 (0.11)	0.58 (0.56)	0.016 (0.019)	**0.0020** **(0.0033)**	**0.0039** (0.0066)	0.13 (0.16)	0.64 (0.98)	0.75 (0.64)
Cohen’s d	**1.0**	**2.4**	0.5	0.2	**1.0**	**0.9**	**0.8**	0.3	0.0	0.1
ANOVA p-value (Loko vs. CGT)	0.661	0.628	0.573	0.708	0.102	0.430	0.903	0.973	0.777	0.165
p value of regression with dose	0.41	**0.04**	N/A	N/A	0.43	0.86	0.18	N/A	N/A	N/A

^1^ANOVA p-value row refers to the post-MANOVA individual outcome comparisons between CGT and Lokomat®

^2^“p-value Loko WSR (t-test)” and “p-value CGT WSR (t-test)” are the Wilcoxon Signed Rank test using one-sample (i.e. comparing means of pre vs. post change scores to zero)

^3^Statistical analysis excludes participants who withdrew from study

^4^For FAC only, the ANCOVA was computed with number of training sessions (i.e. dose) as the covariate. The partial eta squared (η^2^) was 0.17; p value comparing means between groups was 0.64

^a^Not included in the change score data was that 5 of the 9 participants, combined across both groups, could not complete the STS task at admission but were able to complete it at discharge. The intake completion time of these participants was not defined (or infinity) and so their change scores could not be calculated. The data of these 9 participants did not contribute to the statistical evaluations (WSR, MANOVA or the subsequent post-hoc comparisons). The individual times were: 12s (CGT), 17s (CGT), 36s (CGT), 16s (CGT), 37.7 (Lokomat®)

## Discussion

It was feasible and well-tolerated to incorporate an additional three hours per week of gait training therapy into all participants’ schedules over a large portion of their inpatient stay. On average, study participants received 11.3 supplemental therapy sessions (12.8 CGT, 9.8 Lokomat®). It has been noted that other aspects of each individual’s plan of care and stage of recovery can be an obstacle to supplemental therapy [19] but with a clear focus and charge to incorporate additional gait training, finding three extra hours per week proved to be practically achievable during early post stroke inpatient stay time for this small cohort. This was completed during “normal care hours” and did not require night or weekend time to achieve. It is worth noting that current mandates are that IRF stroke patients receive at least three hours of therapy per day, five days a week during their inpatient stay. This therapy is divided between physical, occupational and speech therapies – in our institution, at about 1.5hours, 1 hour and 0.5 hours, respectively. The physical therapy is then split, as needed, to address functional limitations and goals as appropriate for each patient. As such, three hours of additional gait training per week represents a significant increase over a patient’s existing therapy regimen.

Enrolled participants tolerated additional exercise well. The cardiorespiratory inclusion criteria for study participants biased us to expect this. There was a slight reduction in therapists' perceived exertion in the entire cohort over the course of the study. Neither individual treatment group analysis showed statistical significance given the small group size, the relatively small change (0.7 for each group) and the relatively high variability (1.1 Lokomat®, 2.4 CGT) of that change within each group. The Lokomat® group showed a medium effect size (0.62).

Receiving *supplemental* therapy of either form led to marked improvement across many of the study measures (p<0.005 in 9 of 20 comparisons, Table 2). To further support this, we compared study data to data from our reference group who did not receive supplemental training. This large group of 415 diverse patients had an average age of 53.1 years (standard deviation, 5.1 years), were 60% male, and 85% had an ischemic stroke (15% hemorraghic). These data suggest a fairly similar group to the current study group – though not a true matched control. They were, overall, slightly younger than the entire study cohort but about the same age as the CGT group (both of whom were about 10 years younger than the Lokomat® group), slightly less %male (60% male vs. 73% for the study group), and slightly greater % ischemic stroke (85% vs. 73% for the study group). This reference group had a higher average intake FIM motor score (31) and a similar average discharge score (51.5) for an average improvement of about 20.5. In contrast, the study participants’ FIM scores improved 24.5 for the Lokomat® group and 26.6 for the CGT group, both representing a meaningful increase over the reference group FIM improvement [20]. As such, our findings support that the supplemental gait training was valuable to improve mobility function at discharge based on FIM outcome measure.

The reference group having higher intake average FIM score can be viewed in two ways. First, having higher function may allow them to achieve more during gait training. Alternatively, the lower the intake score, the more *potential room* for improvement. The latter idea is consistent with the general finding that lower functioning (non-ambulatory) participants obtained the greatest benefits from electromechanical assist in training [21] and supported by findings such as change in self-selected velocity (SSV) being negatively correlated to SSV at intake [22]. Also, the stroke subtype has commonly been thought to affect functional prognosis where ischemic stroke patients tend not to improve as well during therapy. Recent work suggests that this may not be true – and so perhaps this discrepancy in our reference and research cohorts is not so important [23–25]. Some of these factors could be contributing to differences observed in outcomes between our reference and study groups. However, our data clearly indicate that both groups improved considerably during their inpatient rehabilitation stay and, at least in the comparison we were able to make, that improvement was likely, at least in part, due to the supplemental training they received.

One additional benefit of the supplemental therapy was that both groups showed an *absence* of a decrease in the PROM and an *absence* of an increase in spasticity (MAS) of the paretic side ankle and knee joints (Table 2, Ankle DF, Knee Ext, Ank MAS, Knee MAS columns were not statistically different (admission vs. discharge) for both groups). Reduction in PROM is known to occur immediately post-stroke [26] due to development of an upper motor neuron syndrome, the resulting spasticity, reduced motor control and activity limitations. Preventing this decline can contribute to lessened functional limitations both immediately and long-term. PROM was found not decreased for the ankle (Lokomat®: mean, µ=+4.4 degs, WSR p=0.77, CGT: µ=+1.3, WSR p=0.64) and for the knee (Lokomat®: µ=+1.3 degs, WSR p=0.25; CGT: µ=− 0.3 degs, p=0.75). Note, a positive value (i.e. +4.4) indicates the PROM increased slightly, so for both groups, ankle PROM and for the Lokomat® group knee PROM actually increased slightly. Spasticity, gauged by MAS, slightly increased (not statistically significant) for ankle in both groups and knee in the CGT group. Increases were modest, between 0.2 and 0.4, on average per group.

Outcome variables linked to functional gains showed the majority of the improvements in both groups. FAC, FIM, 5xSTS, 2MWT, and 10MWT showed large effect sizes (>0.8) in both groups – as well as a trend towards, though not statistically significant, differential effect between groups. FAC and FIM showed average improvements of greater than 1.25 and almost 25, respectively. The average FAC at admission was between 1 and 2, indicating participants required continuous support for weight bearing/balance or intermittent support with balance/coordination during walking. The average score at discharge was just below 3, indicating the requirement of physical support for walking was mostly eliminated. As noted previously, the 5×STS times also improved considerably. There was a notable differential in improvement in STS time between the groups. The Lokomat® group improved by just over 50% (45s at admission vs. 22s at discharge) whereas the CGT group improved by just under 30% (29s at admission to 21s at discharge). We wish to highlight that 9 participants could not complete the STS task at admission and 5 of these could at discharge (see note a). Though these data did not contribute to above reported % changes and statistical analyses, their functional gains were just as important, if not more so, than being able to stand *more quickly* – as was reported for the rest of the cohort. The 2MWT also showed similar marked improvements. The entire cohort, on average, was able to quadruple walking distance from just under 10m at admission to slightly over 40m at discharge. The difference is even more striking when segregated by intervention. The Lokomat® group improved nearly ten-fold, from 3m at admission to 29m at discharge and the CGT group more than tripled (16m at admission to 56m at discharge). A similar picture emerged with the 10MWT. Though the overall group change in walking velocity was 0.24m/s, the Lokomat® group went from 0.02m/s to 0.26m/s and the CGT group went from 0.17m/s to 0.40m/s. Improvements in both groups are substantial and clinically meaningful[27]. The Lokomat® group improvement by 50% in 5×STS time and near 10-fold for 2MWT distance and 10MWT velocity are remarkable and prompted post-hoc analysis of differential effect in modality.

Overall, an important point this work helped to establish is that standard of care plus supplemental therapy can lead to many desired improvements and lack of decline, and this can be done in a fairly efficient manner during the inpatient stay without undue burden on therapist (vis-à-vis reduced RPE scores). Additional studies need to be done to further refine the supplemental protocol, including defining optimal intensity and duration, patient demographics as well as delivery mode. The use of robotics can be an important factor in the practical implementation of supplemental therapy as trying to balance therapist time and physical demands with increasing dosage, timing and scheduling during increasingly shorter inpatient stays can be challenging.

Based on the above noted possible differences in effects between treatment groups, we formally explored whether there was a statistical effect of modality. A post-hoc overarching statistical analysis of robotic vs. conventional (CGT) therapy did not show a statistically significant differential effect (MANOVA p=0.21, Pillai Trace). We compared only the interval variables (ankle and knee PROM, 2MWT, 10MWT and 5xSTS) using a single factor MANOVA with post-hoc ANOVAs to compare differences in individual variables. Only these 5 variables were compared since a MANOVA analysis ***requires*** interval variables. Though not significant, two of these five variables did show medium to *nearly* large effect sizes (Cohen’s *d* of 0.56 for knee PROM and 0.77 for the 5xSTS). Medium to large effect sizes yet not significant p-value (MANOVA) may be due to the relatively small sample size and short duration of the intervention (up to an extra three hours per week for approximately three weeks). Because of the relatively large differences in dose between groups, the regression analysis was also evaluated for FIM, FAC, 10MWT, 2MWT and 5xSTS. Only the FAC was significantly correlated with dose (Table 2, last row). Thus, only for FAC did the covariate have a significant effect. For FAC only, the ANCOVA was computed with number of training sessions as the covariate (Table 2, Note #4). The partial eta squared (h^2^) value of 0.17 indicates that dose accounted for only 17% of the variance in the FAC score. The adjusted means did grow more different, supporting that the Lokomat® group improved more (mean change of 1.27 vs. 1.06 for CGT group) but still did not reach significance (p=0.64). We acknowledge this trial was not designed or powered to show this effect but felt it important to highlight differences in outcomes between modalities as it has implications for future trials and subsequently how best to administer supplemental therapy.

The potential differential effect favoring robotic therapy is particularly striking given that, though not by design, the Lokomat® group received less overall number of supplemental sessions than the CGT group. The Lokomat® training group had approximately 30% fewer sessions per IRF stay, but the stepping intensity of the gait specific training was higher with this device compared to the CGT intervention. This assessment of increased stepping is *subjective*, based on observation of therapy sessions; increased step count in robotic therapy compared to CGT was not documented in the current work but has been previously shown [19]. CGT was delivered manually by one or multiple therapist – and so required all participants to coordinate efforts to get productive “training” steps to be realized. Lokomat® training was delivered by the robot and so once the setup was accomplished, the gait training component was more time-condensed and is suspected to have resulted in greater overall steps during the allotted time. Preliminary evidence exists that patients with stroke can improve their walking recovery and quality of life when higher doses of aerobic and stepping activity are provided within 1-4 weeks post injury [28]. Our work similarly, albeit subjectively, supports that increased stepping achieved in the Lokomat® (vs. CGT) may be a key factor in improved outcomes with fewer visits.

Recent work, including reviews and meta-analyses of a moderate body of previous work also suggests but does not clearly indicate differential benefits of robotic training for patients in the acute and subacute phases of stroke [21, 29–32]. There appears to be more support for robotic therapy assisting non-ambulatory acute stroke patients [21, 32, 33] or generally more impaired patients [34, 35] or favoring acute/sub-acute as opposed to chronic stroke patients [30, 31, 36] although limited evidence exists that higher functioning ambulatory patients can benefit [8]. Our results add evidence to further the idea that robotic based training is helpful earlier post-stroke but even for patients who are ambulatory at baseline. Just as in our study, combination of robotic and conventional therapy compared to the *same intensity* (usually just meaning duration but not truly intensity) of only conventional found significant improvements in functional ambulation for both training groups, but showed no significant differences between the two intervention groups [37]. That study used mostly different outcome measures than in our study. Gait symmetry and lean body mass improved, however, in the robotic group only. There is growing evidence that combining conventional and robotic modes maybe, in and of itself, a useful approach [23, 31, 32, 38]. Our robotic intervention group actually received this blended therapy as they retained their mandated standard of care that was delivered conventionally while receiving the robotic supplemental training. Overall, optimizing therapy (mode, frequency and dose) needs additional study to help determine for what stage (acute vs. chronic), when specifically during recovery (ex. 30, 60, 90 days post injury, etc.), what functional level (ambulatory vs. not) of stroke patients and how modality can best be used in gait training [10, 39, 40].

## Limitations

Study participants were inpatients and subject to many constraints, not the least of which is how long they were able to remain on the inpatient unit. As such, some factors could not be controlled as closely as would have been ideal. The sample size was small but appropriate to determine feasibility of providing supplemental therapy during the inpatient stay – which was a primary study objective. The study was not powered to fully explore the extent of differences between the two modalities – which would require a much larger cohort and/or more tightly controlled groups, at least for the current study measures. Post-hoc estimates for study size were n=392 (for each arm, assuming a parity enrollment ratio) for the FIM score changes and standard deviations reported (https://clincalc.com/stats/samplesize.aspx). The post-hoc reference group who did not receive supplemental gait training were not as well matched in characteristics as the two study groups were to each other, nor did we have as much of the functional data in eRehabData® as was collected for the study participants. It is noted that the 10MWT and 2MWT both allowed but did not control use of assistive devices. In addition, some participants for whom an AFO was indicated, may have received these devices a few days before discharge. Variability due to both of these factors may have contributed to differences observed in the performances on these tests. These shortcomings aside, the differences observed (between supplemental vs. no-supplemental therapy as well as Lokomat® vs. CGT) were not merely mathematical but functionally meaningful, in our opinions, and had medium and sometimes large effect sizes, despite not always being statistically significant. Several factors circumscribe the generalizability of the present results. Our hospital has had the Lokomat® for many years and has a number of therapists considerably familiar with the device prior to the start of the study. This, along with having a large overall team of therapist, contributed to the practicality of implementing this protocol and finding/adjusting coverage to allow the small amount of supplemental therapy to be provided during regular treatment hours. The participants were very recent stroke patients (<three weeks post injury) and so even other “acute” stroke patients but at different stages of recovery could show different responses to the supplemental treatment. Our population, in part due to inclusion/exclusion criteria, in part due to small sample size, was fairly constrained and not likely fully representative of all recent acute stroke patients. The population demographics of being in a fairly affluent suburban location likely further contributed to differences from a truly national sample of acute stroke patients. The pilot sample size of 30, of course, contributed to this limited representation as well.

## Conclusions

Providing acute stroke patients with additional walking-focused locomotor training is feasible in an IRF environment without undue strain on patient or staff when effort is placed in scheduling such intervention to supplement mandated therapy time. Participants in both intervention groups showed meaningful improvements in most functional outcomes (FAC, FIM, 5xSTS, 2MWT, 10MWT) – compared to their own intake measures (i.e. at the start of their inpatient stay) and a differential improvement in FIM compared to the post-hoc no supplemental therapy group (standard of care). Neither group declined in PROM and spasticity measures. The Lokomat® group showed improvement with fewer number of training sessions but subjectively assessed increased stepping per session. This supports the overall time efficiency of this gait training robotic tool for patients in early stroke rehabilitation. Several functional measures showed practical but not statistical differences between therapy mode. Determining optimal dose of supplemental therapy (of either mode) and whether the Lokomat® group’s progress plateaued or would continue for additional sessions may help to optimize the focus of therapy during this critical phase of acute stroke rehabilitation and should be studied in the future.

### Supplementary Information

**Additional file 1.** Treatment Option Descriptions.

## Data Availability

The datasets used and/or analysed during the current study are available from the corresponding author on reasonable request.

## References

[1] Li S, Francisco GE, Zhou P (2018). Post-stroke hemiplegic gait: new perspective and insights. Front Physiol.

[2] Duncan PW (2005). Management of adult stroke rehabilitation care: a clinical practice guideline. Stroke.

[3] Tsao CW (2022). Heart disease and stroke statistics—2022 update: a report from the American Heart Association. Circulation.

[4] Olney S, Richards C (1996). Hemiparetic gait following stroke. Part I: characteristics. Gait Posture.

[5] Go AS, Roger VL, Benjamin EJ, Berry JD, Blaha MJ, Dai S, Ford ES, Fox CS, Franco S, Fullerton HJ, Gillespie C, Hailpern SM, Heit JA, Howard VJ, Huffman MD, Judd SE, Kissela BM, Kittner SJ, Lackland DT, Lichtman JH, Lisabeth LD, Mackey RH, Magid DJ, Marcus GM, Marelli A, Matchar DB, McGuire DK, Mohler ER, Moy CS, Mussolino ME, Neumar RW, Nichol G, Pandey DK, Paynter NP, Reeves MJ, Sorlie PD, Stein J, Towfighi A, Turan TN, Virani SS, Wong ND, Woo D, Turner MB (2014). Executive summary: heart disease and stroke statistics–2014 update: a report from the American Heart Association. Circulation.

[6] Batchelor FA (2012). Falls after stroke. Int J Stroke.

[7] Raffin E, Hummel FC (2018). Restoring motor functions after stroke: multiple approaches and opportunities. Neuroscientist.

[8] Esquenazi A (2013). A randomized comparative study of manually assisted versus robotic-assisted body weight supported treadmill training in persons with a traumatic brain injury. Pm r.

[9] Schmidt RA, Lee TD (2011). Motor control and learning : a behavioral emphasis.

[10] Morone G (2017). Robot-assisted gait training for stroke patients: current state of the art and perspectives of robotics. Neuropsychiatr Dis Treat.

[11] Cooke EV, et al. The effects of increased dose of exercise-based therapies to enhance motor recovery after stroke: a systematic review and meta-analysis. (1741–7015 (Electronic)). 10.1186/1741-7015-8-60 PMC2966446 20942915

[12] Kwakkel G (2009). Intensity of practice after stroke: more is better. Schweiz Arch Neurol Psychiatr.

[13] Dombovy ML. Introduction: the evolving field of neurorehabilitation. (1080–2371 (Print)). 10.1212/01.CON.0000399065.23826.f0 22810860

[14] Biernaskie J, Chernenko G, Corbett D (2004). Efficacy of rehabilitative experience declines with time after focal ischemic brain injury. J Neurosci.

[15] Zeiler SR, Krakauer JW (2013). The interaction between training and plasticity in the poststroke brain. Curr Opin Neurol.

[16] Zeiler SR (2019). Should we care about early post-stroke rehabilitation? Not yet, but soon. Curr Neurol Neurosci Rep.

[17] Coleman ER (2017). Early rehabilitation after stroke: a narrative review. Curr Atheroscler Rep.

[18] Liu Y, et al. Early rehabilitation after acute stroke:the golden recovery period. Acta Neurol Taiwan 2022. 34918304

[19] Cao N (2021). Implementing robotic-assisted gait training in acute inpatient stroke rehabilitation: a quality improvement initiative. J Int Soc Phys Rehabil Med.

[20] Beninato M (2006). Determination of the minimal clinically important difference in the FIM instrument in patients with stroke. Arch Phys Med Rehabil.

[21] Mehrholz J (2017). Electromechanical-assisted training for walking after stroke. Cochrane Database Syst Rev.

[22] Moore JL (2020). Implementation of high-intensity stepping training during inpatient stroke rehabilitation improves functional outcomes. Stroke.

[23] Dierick F (2017). Hemorrhagic versus ischemic stroke: Who can best benefit from blended conventional physiotherapy with robotic-assisted gait therapy?. PLoS ONE.

[24] Perna R, Temple J (2015). Rehabilitation outcomes: ischemic versus hemorrhagic strokes. Behav Neurol.

[25] Salvadori, E.A.-O., et al., Comparison between ischemic and hemorrhagic strokes in functional outcome at discharge from an intensive rehabilitation hospital. LID. 10.3390/diagnostics11010038 (2075–4418 (Print)). 10.3390/diagnostics11010038 PMC7824133 33379391

[26] Wissel J (2015). Post-stroke spasticity: predictors of early development and considerations for therapeutic intervention. Pm r.

[27] Tilson JK (2010). Meaningful gait speed improvement during the first 60 days poststroke: minimal clinically important difference. Phys Ther.

[28] Klassen TD (2020). Higher doses improve walking recovery during stroke inpatient rehabilitation. Stroke.

[29] Nedergård H (2021). Effect of robotic-assisted gait training on objective biomechanical measures of gait in persons post-stroke: a systematic review and meta-analysis. J Neuroeng Rehabil.

[30] Meng G (2022). Effect of early integrated robot-assisted gait training on motor and balance in patients with acute ischemic stroke: a single-blinded randomized controlled trial. Ther Adv Neurol Disord.

[31] Lin YN (2022). Hybrid robot-assisted gait training for motor function in subacute stroke: a single-blind randomized controlled trial. J Neuroeng Rehabil.

[32] Mehrholz J (2020). Electromechanical-assisted training for walking after stroke. Cochrane Database Syst Rev.

[33] Pohl M (2007). Repetitive locomotor training and physiotherapy improve walking and basic activities of daily living after stroke: a single-blind, randomized multicentre trial (DEutsche GAngtrainerStudie, DEGAS). Clin Rehabil.

[34] Morone G (2012). Who may have durable benefit from robotic gait training?: a 2-year follow-up randomized controlled trial in patients with subacute stroke. Stroke.

[35] Morone G (2011). Who may benefit from robotic-assisted gait training? A randomized clinical trial in patients with subacute stroke. Neurorehabil Neural Repair.

[36] Louie DR, Eng JJ (2016). Powered robotic exoskeletons in post-stroke rehabilitation of gait: a scoping review. J Neuroeng Rehabil.

[37] Husemann B (2007). Effects of locomotion training with assistance of a robot-driven gait orthosis in hemiparetic patients after stroke: a randomized controlled pilot study. Stroke.

[38] Nolan KJ (2021). Utilization of robotic exoskeleton for overground walking in acute and chronic stroke. Front Neurorobot.

[39] Mehrholz J (2013). Electromechanical-assisted training for walking after stroke. Cochrane Database Syst Rev.

[40] Schröder J (2019). Feasibility and effectiveness of repetitive gait training early after stroke: A systematic review and meta-analysis. J Rehabil Med.

