# Supplementary material for: Feasibility and outcomes of supplemental gait training by robotic and conventional means in acute stroke rehabilitation
Source: J Neuroeng Rehabil. 2023 Oct 4;20:134. doi: 10.1186/s12984-023-01243-3 (PMC10552424; doi:10.1186/s12984-023-01243-3)
Supplement: Supplementary file 1 — Additional file 1. Treatment Option Descriptions. [file 12984_2023_1243_MOESM1_ESM.docx]

**Conventional Treatment:**

The activity structure followed this scheme:

1. If unable to stand or ambulate patient participated in activities in the following priority order:
   - lower limb stretching, strengthening, bed mobility activities with attempt to progress
   - sitting balance from supported to unsupported with attempt to progress.
2. If able to stand but not ambulate, patient participated activities in the following priority order:
   - transfer training across surfaces of different heights using stand pivot,
   - standing balance training to increase endurance and initiate pre-gait activities,
   - pre-gait activities training, weight shifting and limb loading,
   - ambulation training with assistance with walker, supported walker, hemi-rail with attempt to progress as clinically indicated.
3. If able to walk, patients participated in walking activities in the following priority order:
   - maximizing number of steps.
   - walking for the duration of the session.
   - minimizing dependence on therapist

**Lokomat™ Treatment:**

The activity structure followed this scheme:

1. If subjects were not ambulatory, they participated in activities in the following priority order:
   - maximize the time they could tolerate being upright in the device, with progression to being ranged in the device.
   - once able to tolerate being upright in the device for at least 10 minutes, subjects were encouraged to step with the device if they were able, with the goal of maximizing number of steps.
2. If subjects were ambulatory, they participated in activities in the following priority order:
   - walking for the duration of the session with the maximum number of steps.
   - walking for as much of the session as possible with maximum number of steps
   - walking with as little guidance force as possible
   - walking with as little BWS as possible
   - *Notes on the above priorities and how training was adjusted to meet them:*
     1. *Speed was chosen to match their overground walking speed at the start of the session. Speed was not raised above daily overground speed.*
     2. *Guidance force was initially set to 50% with a goal to reducing it, if it could be done without reducing active time and number of steps (+/- 10% was considered constant).*
     3. *BWS was adjusted downward* *if could be done* *without reducing active time and number of steps (+/- 10% was considered constant)*
